# Speech Differences between Multiple System Atrophy and Parkinson's Disease

**DOI:** 10.1002/mdc3.70094

**Published:** 2025-05-03

**Authors:** Tom Hähnel, Anna Nemitz, Katja Schön, Luise Berger, Annemarie Vogel, Doreen Gruber, Nils Schnalke, Stefan Bräuer, Björn H. Falkenburger, Florin Gandor

**Affiliations:** ^1^ Department of Neurology, Medical Faculty and University Hospital Carl Gustav Carus TUD ‐ Dresden University of Technology Dresden Germany; ^2^ Movement Disorders Clinic Kliniken Beelitz GmbH Beelitz Germany; ^3^ Department of Neurology Otto‐von‐Guericke University Magdeburg Magdeburg Germany; ^4^ German Center for Neurodegenerative Diseases (DZNE) Dresden Germany; ^5^ Department of Neurology Macquarie University Sydney Australia

**Keywords:** Parkinson's disease, multiple system atrophy, speech, dysarthria, voice

## Abstract

**Background:**

Delineation of Parkinson's disease (PD) from multiple system atrophy (MSA) can be challenging in early disease stages. Speech characteristics have been studied as digital biomarkers in PD and ataxias. Currently, data on speech in MSA is limited.

**Objectives:**

To determine whether speech characteristics can serve as a digital biomarker to differentiate between MSA and PD.

**Methods:**

Twenty‐one MSA patients and 23 PD patients underwent a battery of speech assessments: text reading, sustained phonation and diadochokinetic tasks. Speech characteristics were extracted using the software, Praat.

**Results:**

MSA and PD speech can be described by three meaningful factors. MSA speech exhibited more reading pauses, higher pitch variability, prolonged syllables, and a more irregular speech rhythm, allowing differentiation from PD with a ROC‐AUC of 0.89. Speech characteristics were correlated with motor impairment and disease severity.

**Conclusion:**

MSA can be differentiated from PD with good accuracy using speech analysis.

Multiple System Atrophy (MSA) is a progressive, neurodegenerative disease presenting with parkinsonism and/or cerebellar symptoms in combination with dysautonomia. Accuracy of MSA diagnosis is especially low at initial consultation and thereby delays or even prevents the enrollment of MSA patients in clinical trials that examine potentially disease modifying treatments in the early disease course.^1^

Speech characteristics have been widely studied as a digital biomarker in PD^2^ and ataxias.^3^, ^4^, ^5^, ^6^, ^7^ In contrast, speech data in MSA is limited and only available from small cohorts.^8^, ^9^, ^10^, ^11^, ^12^, ^13^, ^14^ In addition, the majority of studies are from native Czech speakers.^8^, ^10^, ^11^, ^14^ While several studies have demonstrated the transferability of speech analysis across languages in hypokinetic dysarthria,^15^, ^16^, ^17^ it remains unclear whether this applies to MSA, where dysarthria exhibits a mixed pathology of spastic, ataxic, and hypokinetic characteristics. Thus, it remains unclear if speech characteristics can serve as a robust digital biomarker for differentiating between MSA and PD.

Because PD has a high prevalence of hypokinetic speech,^2^ there is a considerable clinical overlap with MSA regarding speech impairment.^11^ In contrast to PD, MSA shows a faster disease progression^18^ and lacks levodopa responsiveness of Parkinsonian symptoms.^19^, ^20^ Thus, MSA patients typically have a higher motor impairment and disease burden with shorter disease duration compared to PD.^18^ It is therefore essential to account for differences in motor impairment, which has not been included in previous studies.^8^, ^9^, ^10^, ^11^, ^12^, ^13^, ^14^, ^15^

In this work, we analyze speech recordings of patients with MSA and PD. We investigate the factors of speech that characterize both diseases and develop statistical models to delineate MSA from PD. In addition, we analyzed the correlation of speech characteristics with (I) motor impairment as measured by the Movement Disorders Society's Unified Parkinson's Disease Rating Scale (MDS‐UPDRS) part III,^21^ and (II) the severity of MSA as measured by Unified Multiple System Atrophy Rating Scale (UMSARS).^22^

## Methods

### Recruitment of Study Participants

Between March 2021 and November 2022, 23 people with PD according to the MDS diagnostic criteria for PD^23^ and 21 people with probable or possible MSA according to the second consensus statement on the diagnosis of MSA^24^ were recruited consecutively at two German movement disorders centers. Written informed consent was obtained from all participants. All participants were on stable dopaminergic treatment and were assessed in the ON state. MDS‐UPDRS III^21^ and UMSARS were obtained.^22^ Levodopa equivalent daily doses (LEDD) were calculated using the current recommendations.^25^

### Speech Recordings

Recordings were performed in a quiet room with low ambient noise using an Olympus Linear PCM Recorder LS‐P4 and a mouth‐to‐microphone distance of 5 cm. The recordings were processed at 16‐bit resolution and 44.1 kHz sample size. Artificial recording alterations such as equalization, automatic gain control, and noise reduction were disabled.

### Speech Analysis

The following tasks were recorded^1^: sustained phonation (SP) of the vowels /a/ and /i/, the two diadochokinetic tasks (DDK)^2^ sequential motion rates /pΛtΛkΛ/ and^3^ alternating motion rates /pΛ/ and /tΛ/, and^4^ text reading (R). Recordings for each task were performed twice and closely followed the *Guidelines for Speech Recording and Acoustic Analysis in Dysarthrias of Movement Disorders*.^26^ A set of 22 speech characteristics was calculated using Praat.^27^ Visual representations (Fig. S1) and detailed descriptions (Table S1, Supplemental File S1) of speech factors together with typical MSA (Supplemental File S2) and PD speech recordings (Supplemental File S3) are available in the supplement. Sex‐specific analyses are provided in Supplemental File S1 (Fig. S2).

### Statistical Analyses

Exploratory factor analysis and t‐distributed Stochastic Neighbor Embedding (t‐SNE) was used to investigate the factors describing dysarthria in PD and MSA. MSA and PD speech characteristics, MDS‐UPDRS III, age, and disease duration were compared using t‐test, Welch's test or Mann–Whitney‐U test, depending on the distribution of the data. Sex and dysarthria subtypes were compared using Fisher's exact tests, Hoehn & Yahr (H&Y) and dysarthria severity using Kolmogorov–Smirnov tests. Additionally, MSA and PD speech characteristics were compared using a linear model with MDS‐UPDRS III as covariate. Observations with an absolute z‐score greater than 3 were removed to limit the effect of outliers. Logistic models were used to differentiate MSA from PD. Details are provided in Supplemental File S1.

### Code Availability

The complete Praat scripts used for our analyses are available at github.com/t-haehnel/MSA-Speech-Analysis-Praat.

## Results

### Characteristics of Study Participants

Speech recordings from 21 MSA patients and 23 PD patients were included in this study. Text reading recordings were available for all patients. Diadochokinetic tasks were recorded in 13 MSA patients and 22 PD patients. Sustained phonation was recorded in all PD patients and 13 MSA patients. Based on these recordings, we calculated 22 speech characteristics described in Table S1. Clinical measurements were available for all patients (MDS‐UPDRS III) and 16 MSA patients (UMSARS).

Age and sex distribution did not differ significantly between groups (MSA: 67.2 years, 43% male; PD: 70.4 years, 70% male). MSA patients had a shorter disease duration (4.2 years vs 9.8 years, *p* = 0.001), but higher motor impairment as reported by MDS‐UPDRS III (43 vs 29, *p* = 0.001) and the Hoehn & Yahr scale (IV vs II, *p* < 0.001). They were treated with higher LEDD (936 mg vs 623 mg, *p* = 0.01). The median dysarthria severity as reported by the MDS‐UPDRS III item 3.1 was not significantly different between MSA and PD (2 points vs. 1 point). Hypokinetic dysarthria was also the most common perceptual dysarthria subtype in MSA (52%, Table S2). The mean UMSARS (sum of parts I and II) was 48.5 points in the MSA group.

### Speech Factors

We found three common factors underlying speech in MSA and PD: (I) a *time and pauses factor* characterized by the speech characteristics *total pause duration*, *number of pauses*, and *reading duration*; (II) a *harsh voice factor* characterized by the speech characteristics *shimmer*, *HNR* and *jitter*; and (III) a *mixed speech characteristics factor* described by the speech characteristics *mean pause duration*, *syllable count*, and *syllable* duration (Fig. S3/4).

### Speech Characteristics as Marker of Motor Impairment and Disease Severity

Motor impairment as measured by MDS‐UPDRS III was correlated with higher *F0 variability* (perceptually: unstable pitch) in the sustained phonation task and higher *rhythm instability* (perceptually: varying syllable length) in the diadochokinetic task for MSA patients. Overall disease severity in MSA measured by UMSARS part I and II was correlated with higher *intensity variability* (perceptually: unstable loudness) in the sustained phonation task, slower speech (longer *reading duration* and lower *syllable count*), and more pauses (higher *number of pauses* and longer *total pause duration*).

**Figure 1:**
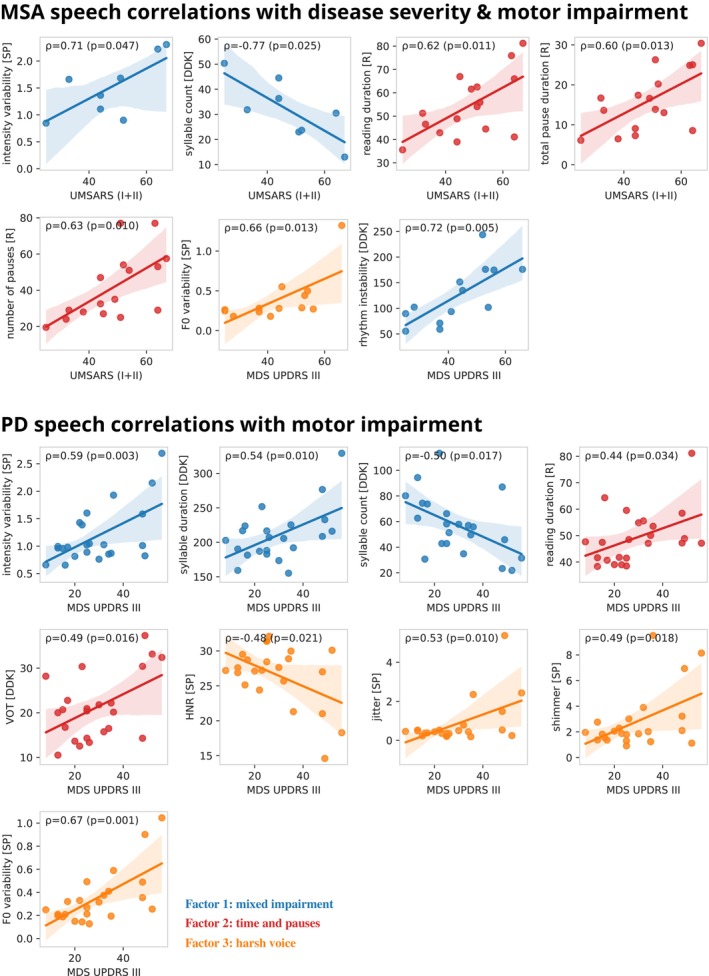
Speech characteristics as markers of motor impairment and disease severity. Significant correlations of speech characteristics with motor impairment (MDS‐UPDRS III; MSA and PD) and disease severity (UMSARS; MSA) with correlation coefficients, *P*‐values and 95% confidence intervals.

Speech characteristics of PD patients also exhibited several correlations with MDS‐UPDRS III (Fig. 1).

### Differentiating MSA and PD Using Speech Characteristics

**Figure 2:**
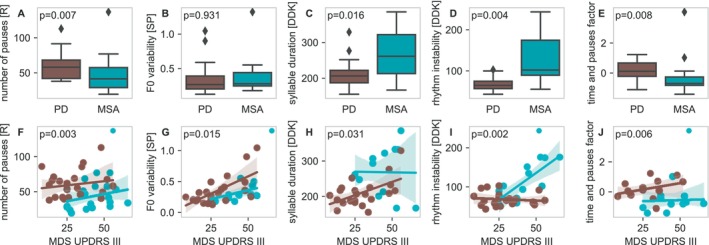
Speech differences between MSA and PD. Speech characteristics and factors with significant differences between MSA (blue) and PD (brown). Univariate comparison (top) and comparisons corrected by MDS‐UPDRS III (bottom) are shown

When analyzing speech differences between MSA and PD without including MDS‐UPDRS III correction, we identified significant differences in several speech characteristics from all speech tasks and all speech factors (Fig. S5–S8, Table S3). However, this analysis is likely to be biased by the higher motor impairment in the MSA group reported by MDS‐UPDRS III (Table S2) in combination with several correlations of speech characteristics with MDS‐UPDRS III (Fig. 1). Therefore, we implemented MDS‐UPDRS III as correction factor. Using this correction, we identified four voice characteristics and one speech factor that were significantly different between MSA and PD (Fig. 2, Table S3). After correction for motor impairment, MSA patients exhibited a higher *rhythm instability* (*p* = 0.002) and longer *syllable duration* (*p* = 0.03) in the diadochokinetic task, and lower *number of pauses* (*p* = 0.003) in the reading task compared to PD. MSA patients presented a lower corrected *F0 variability* for the sustained phonation task (*p* = 0.01) which was not visible in the univariate analysis before. We observed a significant difference with a lower *time and pauses factor* (*p* = 0.006) in the MSA cohort.

Finally, we investigated the potential of our findings to differentiate between MSA and PD based on the four speech characteristics and one speech factor we identified. The best discrimination was observed for *syllable duration* of the diadochokinetic task with an AUC‐ROC of 0.89 (including MDS‐UPDRS III). All models improved after inclusion of MDS‐UPDRS III (Fig. S9).

## Discussion

### Speech Differences between MSA and PD

This study presents the first analysis of speech characteristics in German‐speaking MSA patients, expanding previous knowledge from Czech and Italian MSA cohorts^8^, ^10^, ^11^, ^13^, ^14^ and demonstrating consistent speech characteristics across languages. While higher *rhythm instability*, longer *syllable duration*, and *lower F0 variability* in MSA compared to PD have been described before, we report for the first time that MSA patients exhibit a lower *number of pauses*. We achieved high accuracy in distinguishing MSA from PD, particularly with *syllable duration* in the diadochokinetic task (AUC‐ROC of 0.89), comparable to the performance reported by others.^13^

### Speech Factors

We identified two main speech factors with high internal consistency and clinical interpretability: *time and pause characteristics* and *harsh voice*. Our findings differ from the traditional classification of dysarthria into hypokinetic, ataxic, and spastic domains,^8^, ^11^, ^13^, ^28^ as hypokinetic, ataxic, and spastic speech characteristics were distributed across all three speech factors. Furthermore, most of the speech characteristics traditionally interpreted as hypokinetic did not show any correlation with MDS‐UPDRS III. In contrast, speech characteristics traditionally interpreted as ataxic showed significant MDS‐UPDRS III correlations. A more detailed explanation including several examples is provided in Supplemental File S1.

### Speech Characteristics as Marker of Motor Impairment and Disease Severity

Our study adds evidence to the current, still controversial literature,^29^, ^30^, ^31^, ^32^, ^33^, ^34^, ^35^ that several speech characteristics are a marker of motor impairment in PD. We showed for the first time that speech characteristics report disease severity and motor impairment in MSA, emphasizing the value of acoustic speech analysis for disease monitoring.

### Motor Impairment Correction in Speech Analysis

Our study highlights the importance of correcting for motor impairment when analyzing speech differences between MSA and PD and suggests that previous studies without this correction may have overestimated the impact of some speech characteristics in discriminating between both diseases.^8^, ^9^, ^10^, ^11^, ^12^, ^13^, ^14^ The four speech characteristics that remained significant after correction for motor impairment should be prioritized for further research.

### Limitations

While our sample size is comparable to other acoustic studies,^8^, ^9^, ^10^, ^11^, ^12^, ^13^ the small cohort limits the detection of subtle speech differences. In addition, only 13 of 21 MSA patients completed all of the speech tasks and UMSARS measurements were missing for some MSA patients. Motor impairment in MSA was higher and disease duration was shorter. While this reflects the faster disease progression in MSA,^20^ no definite conclusions can be drawn on whether speech analysis can differentiate both diseases in early stages. Future studies could therefore focus on speech analyses in possible prodromal MSA.^36^

Although all patients were on stable dopaminergic treatment, it remains unclear whether speech analysis should be conducted in the ON or OFF state. Differences in dopaminergic responsiveness may confound results by affecting hypokinetic speech characteristics, potentially impacting diagnostic accuracy by unmasking disease‐specific differences.

Furthermore, jitter and shimmer, which require high‐fidelity equipment and periodic type I or II signals, may be invalid in patients with movement disorders due to impaired periodicity and do not necessarily reflect voice quality in connected speech.^37^, ^38^, ^39^ Further research should focus on other speech parameters less affected by these confounders.

## Conclusion

Our study provides a differential analysis of the speech characteristics in MSA and PD. As shown here, speech analysis could be useful in differentiating MSA from PD.

## Author Roles

(1) Research project: A. Conception, B. Organization, C. Execution; (2) Statistical Analysis: A. Design, B. Execution, C. Review and Critique; (3) Manuscript: A. Writing of the first draft, B. Review and Critique

T.H.: 1A, 1B, 1C, 2A, 2B, 3A.

A.N.: 1C, 2B.

K.S.: 1C.

L.B.: 1C.

A.V.:1C.

D.G.: 1A.

N.S.: 1C, 3B.

S.B.: 1C, 3B.

B.H.F.: 1A, 1B, 2C, 3B.

F.Ga.: 1A, 1B, 2C, 3B.

## Disclosures

**Ethical Compliance Statement:** The study was approved by the institutional review board of Technische Universität Dresden, Germany (BO‐EK‐149032021, BO‐EK‐47012020) and the Brandenburg State Medical Association (S21(a)/2017). Written informed consent was obtained from all participants. We confirm that we have read the Journal's position on issues involved in ethical publication and affirm that this work is consistent with those guidelines.

**Funding Sources and Conflict of Interest:** No specific funding was received for this work. The authors declare that there are no conflicts of interest relevant to this work.

**Financial Disclosures for the previous 12 months:** TH reports a research grant (ParKInsonPredict, 16DKWN1113A) funded by the Federal Ministry of Education and Research of Germany. AN reports a studying grant from the Association of Statutory Health Insurance Physicians Saxony (Kassenärztliche Vereinigung Sachsen). KS declares that there are no additional disclosures to report. LB reports a doctorate grant from Otto‐von‐Guericke University Magdeburg, Germany. AV declares that there are no additional disclosures to report. DG reports royalties from AbbVie Pharma, EverPharma. NS declares that there are no additional disclosures to report. SB declares that there are no additional disclosures to report. BHF reports grants from the German Research Foundation (DFG) and speaker honoraria from AbbVie, Stadapharm, Desitin, Zambon and Bial. FG reports honoraria from AbbVie, BIAL, Merz, Stada, and honoraria from advisory boards for AbbVie, and Stada.

## Supporting information


**File S1.** The file contains additional figures and text presenting methodological details, definitions and explanations of speech characteristics, details of speech tasks and acoustic analysis, the default Praat parameters, speech factor analysis, and sex‐specific analyses.
**File S2.** Audio file of the text reading task from a MSA patient presenting a mixed dysarthria type characterized by hypokinetic (hypophonia, reduced articulation precision, prolonged pauses), ataxic (voice breaks, pitch fluctuations) and spastic/dystonic characteristics (high pitch). The German text and its English translation is given in Supplemental File S1.
**File S3.** Audio file of the text reading task from a PD patient presenting a hypokinetic dysarthria type characterized by typical hypokinetic speech characteristics (hypophonia, reduced articulation precision, prolonged pauses). The German text and its English translation is given in Supplemental File S1.
**TABLE S1.** List of speech characteristics calculated for the different speech tasks.
**TABLE S2.** Clinical characteristics of the MSA and PD cohorts.
**TABLE S3.** Summary of speech characteristics findings.

## Data Availability

Speech recordings cannot be made available due to privacy restrictions. All Praat scripts used for our analyses are available at https://github.com/t-haehnel/MSA-Speech-Analysis-Praat under the MIT license.
