# Supplementary material for: Speech Differences between Multiple System Atrophy and Parkinson's Disease
Source: Mov Disord Clin Pract. 2025 May 3;12(9):1391–6. doi: 10.1002/mdc3.70094 (PMC12481427; doi:10.1002/mdc3.70094)
Supplement: Supplementary file 1 — File S1. The file contains additional figures and text presenting methodological details, definitions and explanations of speech characteristics, details of speech tasks and acoustic analysis, the default Praat parameters, speech factor analysis, and sex‐specific analyses. File S2. Audio file of the text reading task from a MSA patient presenting a mixed dysarthria type characterized by hypokinetic (hypophonia, reduced articulation precision, prolonged pauses), ataxic (voice breaks, pitch fluctuations) and spastic/dystonic characteristics (high pitch). The German text and its English translation is given in Supplemental File S1. File S3. Audio file of the text reading task from a PD patient presenting a hypokinetic dysarthria type characterized by typical hypokinetic speech characteristics (hypophonia, reduced articulation precision, prolonged pauses). The German text and its English translation is given in Supplemental File S1. TABLE S1. List of speech characteristics calculated for the different speech tasks. TABLE S2. Clinical characteristics of the MSA and PD cohorts. TABLE S3. Summary of speech characteristics findings. [file MDC3-12-1391-s001.zip › mdc370094-supitem-0001/mdc370094-sup-0001-TableS1-S3.pdf]

Supplementary Materials

Speech Differences between Multiple System Atrophy and Parkinson's Disease

Tom Hähnel MD[1], Anna Nemitz[1], Katja Schön[3], Luise Berger[2,3], Annemarie Vogel M.Sc.[3], Doreen Gruber[2,3], Nils Schnalke MD[1,], Stefan Bräuer MD[1,4], Björn H. Falkenburger MD[1,4], Florin Gandor MD[2,3,5]

1) Department of Neurology, Medical Faculty and University Hospital Carl Gustav Carus, TUD Dresden University of Technology, Dresden, Germany
2) Department of Neurology, Otto-von-Guericke University Magdeburg, Germany
3) Movement Disorders Clinic, Kliniken Beelitz GmbH
4) German Center for Neurodegenerative Diseases (DZNE), Dresden, Germany
5) Department of Neurology, Macquarie University Sydney, Australia

Corresponding author:
Prof. Dr. med. Florin Gandor
Department of Neurology
Macquarie University Sydney
Suite 204, Level 2
2, Technology Place
Macquarie University 2109
Australia
Mail: florin.gandor@mqhealth.org.au

Additional Tables and Figures

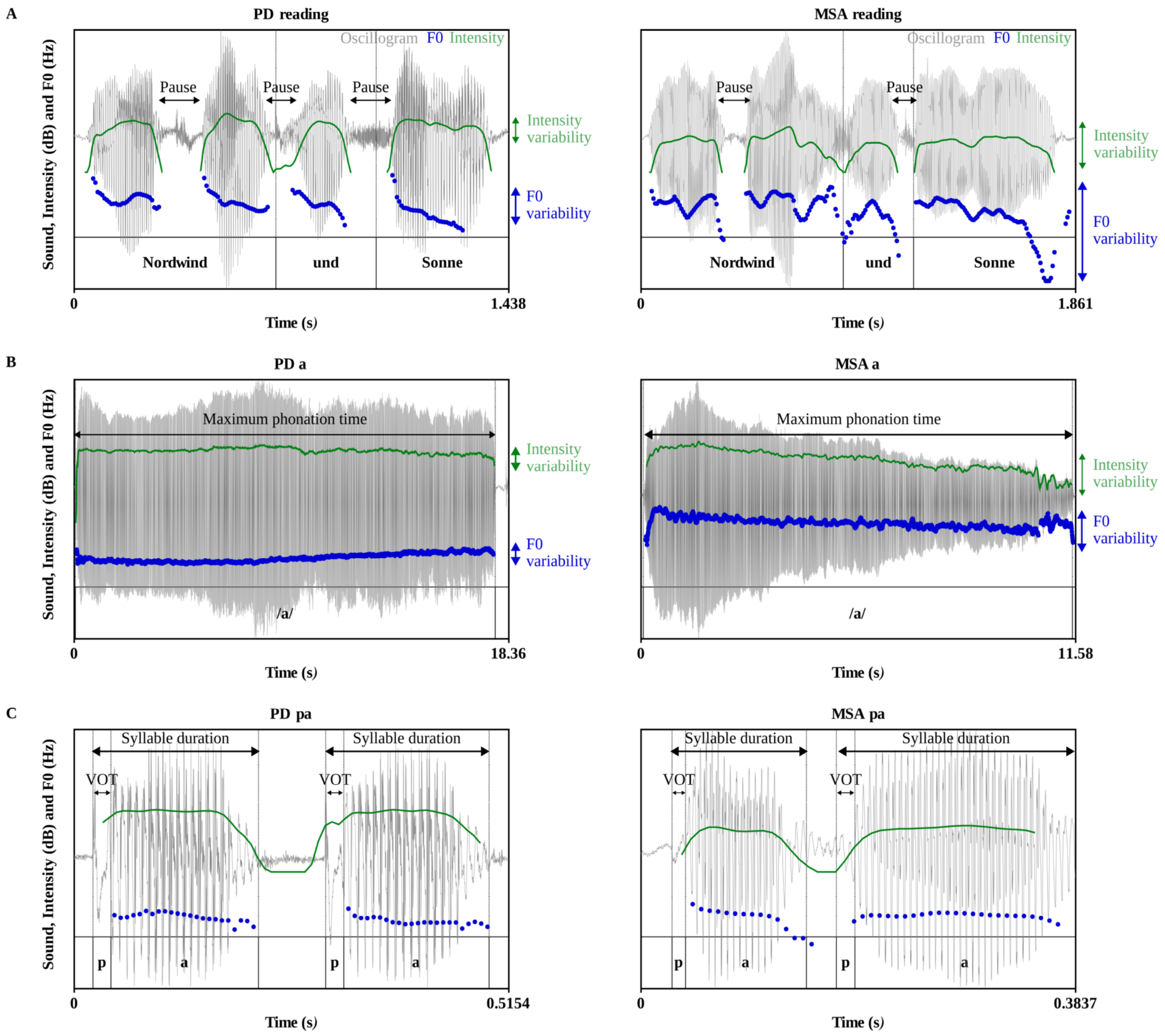

Figure S1: Illustration of speech characteristics for a PD patient and a MSA patient

Speech recordings are shown as oscillograms (gray lines) overlaid with F0 curves (blue) and intensity curves (green) for a PD patient (left column) and a MSA patient (right column). ***A:*** *Reading task: Note the more irregular and fluctuating intensity and F0 curves in the MSA patient, reflecting an ataxic speech pattern. Pauses are also more irregular and sometimes missing (e.g. between "Nordwind" and "und"), resulting in a lower number of pauses. The PD patient shows less variation in intensity and F0 as part of a hypokinetic speech pattern.* ***B:*** *Sustained phonation: Note the more irregular and fluctuating intensity and F0 curves in the MSA patient, reflecting ataxic speech.* ***C:*** *Diadochokinetic task: Note the increased variability of syllable durations in the MSA patient, leading to rhythm instability. Also note the slightly higher variability of voice onset times (VOT), reflecting an ataxic speech pattern. Audio files presenting typical MSA and PD dysarthria are available online.*

Speech characteristic	Speech task	Unit	Definition
**Reading duration**	Reading	s	Total duration required for text reading passage including pauses
**Total pause duration**	Reading	s	Sum of all pause segments with a duration longer than 60 ms. (1)
**Number of pauses**	Reading	-	Number of pauses with a duration longer than 60 ms.
**Mean pause duration**	Reading	ms	Mean duration of pauses with a duration longer than 60 ms.
**Intensity variability**	Reading	dB	Standard deviation of loudness
**F0**	Reading	Hz	Fundamental frequency (F0)
**F0 variability**	Reading	semitones	Standard deviation of fundamental frequency (F0)
**Harmonics-to-noise ratio (HNR)**	Sustained phonation	dB	Degree of acoustic periodicity in the voice signal
**Jitter**	Sustained phonation	%	Period-to-period variability in fundamental frequency (F0)
**Shimmer**	Sustained phonation	%	Period-to-period variability of amplitudes
**Intensity variability**	Sustained phonation	dB	Standard deviation of loudness
**F0**	Sustained phonation	Hz	Fundamental frequency (F0)
**F0 variability**	Sustained phonation	semitones	Standard deviation of fundamental frequency (F0)
**Maximum phonation time (MPT)**	Sustained phonation	s	Total duration of phonation time
**Vowel space area (VSA)**	Sustained phonation	-	Vowel space area calculated as Euclidean distance between the first and second formant of the vowels /a/ and /i/ in the formant space. (2)
**Voice breaks**	Sustained phonation	%	Proportion of subharmonic intervals
**Syllable duration**	Diadochokinetic	ms	Median syllable length
**Syllable count**	Diadochokinetic	-	Number of syllables per diadochokinetic task
**Rhythm acceleration**	Diadochokinetic	‰	Acceleration of syllable duration over the task (in ms acceleration per second speaking duration) (3)
**Rhythm instability**	Diadochokinetic	s	Sum of absolute deviations of each syllable duration from the expected duration divided by the total speech time. The expected syllable duration is calculated by taking the rhythm acceleration into account. (3).
**Voice onset time (VOT)**	Diadochokinetic	ms	Duration from the release of the stop consonant until beginning of voicing
**VOT variability**	Diadochokinetic	ms	Median absolute deviation of the voice onset times

Table S1: List of speech characteristics calculated for the different speech tasks

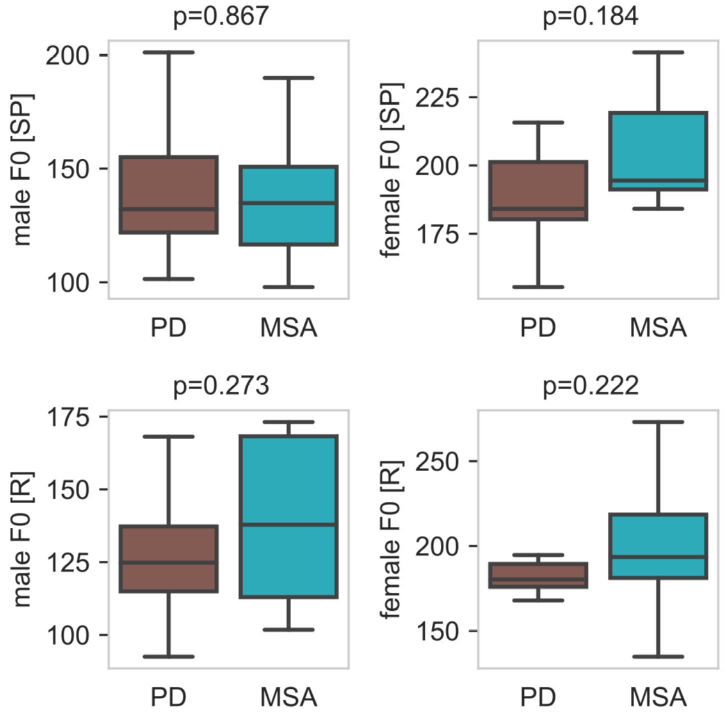

Figure S2: Sex-specific analyses of F0 in MSA and PD
The figure shows the results of univariate sex-specific comparisons of F0 between PD and MSA with corresponding p-values.
Abbreviations: R: reading task, SP: sustained phonation task.

**Clinical characteristics**	**MSA, n = 21 (MSA-C: 6, MSA-P: 15)**	**PD, n = 23**	**MSA vs. PD p-values**
**Age (years)**	67.2 (52 - 83)	70.4 (50 - 88)	0.33
**Sex**	..	..	0.13
female	57 % (12)	30 % (7)	..
male	43 % (9)	70 % (16)	..
**Disease duration (years)**	4.2 (1 - 11)	9.8 (1 - 25)	**0.001**
**MDS-UPDRS III**	42.7 (25 - 66)	29.0 (8 - 56)	**0.001**
**MDS-UPDRS III Speech Item**	..	..	0.56
0	0 % (0)	22 % (5)	..
1	43 % (9)	39 % (9)	..
2	29 % (6)	30 % (7)	..
3	19 % (4)	9 % (2)	..
4	10 % (2)	0 % (0)	..
**Dysarthria subtype**	..	..	**<0.001**
normal	5 % (1)	26% (6)	..
hypokinetic	52 % (11)	74 % (17)	..
ataxic	0 % (0)	0 % (0)	..
spastic	0 % (0)	0 % (0)	..
mixed	43 % (9)	0 % (0)	..
**Hoehn & Yahr**	..	..	**<0.001**
I	0 % (0)	0 % (0)	..
II	5 % (1)	61 % (14)	..
III	33 % (7)	26 % (6)	..
IV	43 % (9)	9 % (2)	..
V	19 % (4)	4 % (1)	..
**UMSARS I**	25.4 (14 - 34)	..	..
**UMSARS II**	23.1 (11 - 33)	..	..
**UMSARS IV**	3.2 (2 - 4)	..	..
**LEDD (mg)**	936 (250-1800)	623 (250-1250)	**0.01**

Table S2: Clinical characteristics of the MSA and PD cohorts

Mean and range are shown for age, disease duration, MDS-UPDRS III, UMSARS, and LEDD. Relative and absolute frequencies are shown for sex, Hoehn & Yahr, MDS-UPDRS III speech item, and dysarthria subtype.

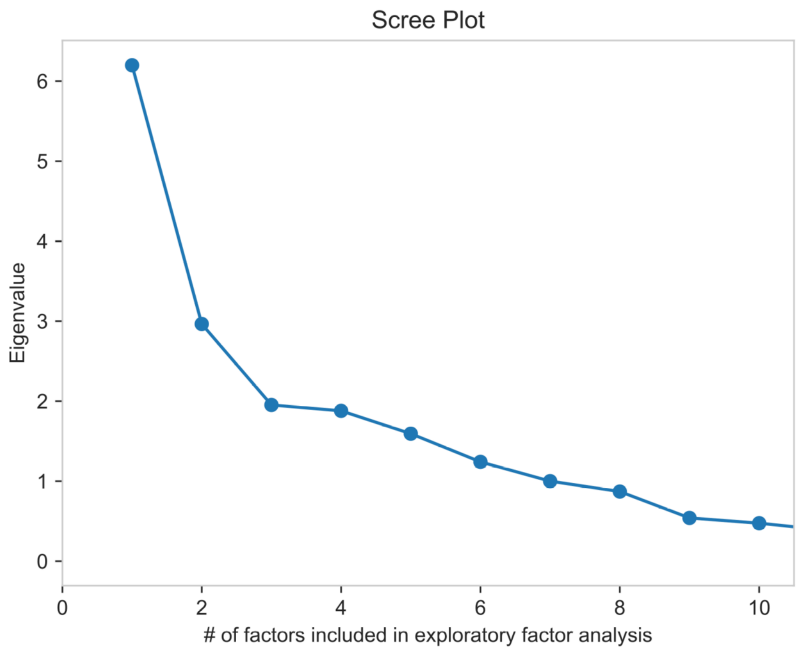

Figure S3: Scree plot for exploratory factor analysis of speech characteristics

Scree plot showing the Eigenvalues for the exploratory factor analysis for 1 up to 10 factors. Based on this plot, k=3 factors were chosen as appropriate.

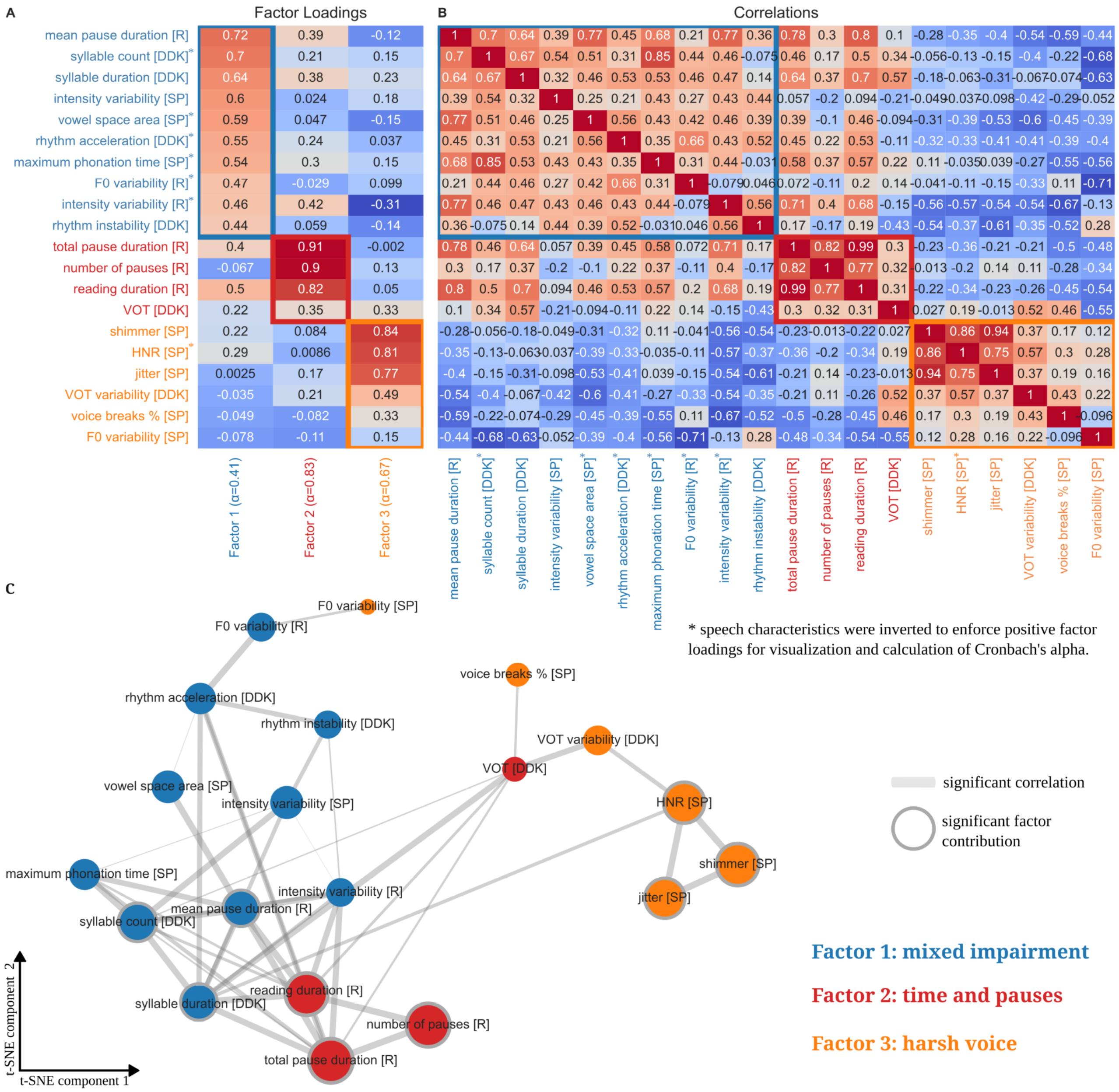

Figure S4: MSA and PD speech factors and correlations between speech characteristics
A: *Factor loadings for speech characteristics. The speech characteristics which are mostly determined by the same factor are surrounded by a colored rectangle. Cronbach's alpha is displayed below each column.* ***B:*** *Correlation heatmap between speech characteristics sorted by the loadings of each factor. Speech characteristics belonging to the same factor are surrounded by a colored rectangle.* ***C:*** *Visualization of correlations between speech characteristics using t-distributed stochastic neighbor embedding (t-SNE). Using t-SNE, speech characteristics with similar correlation patterns are placed closer together while speech characteristics with different correlation patterns are placed further apart. The size of the circle is determined by the absolute value of the factor loading (higher factor loading = larger circle). Speech characteristics considered relevant (factor loading >0.63) are surrounded by a gray circle. Significant (p<0.05) correlations between speech characteristics are indicated by a gray line, while thicker lines indicate a higher absolute value of the correlation coefficient. Abbreviations: DDK: diadochokinetic task, HNR: harmonics-to-noise Ratio, R: reading Task, SP: sustained phonation task, VOT: voice onset time.*

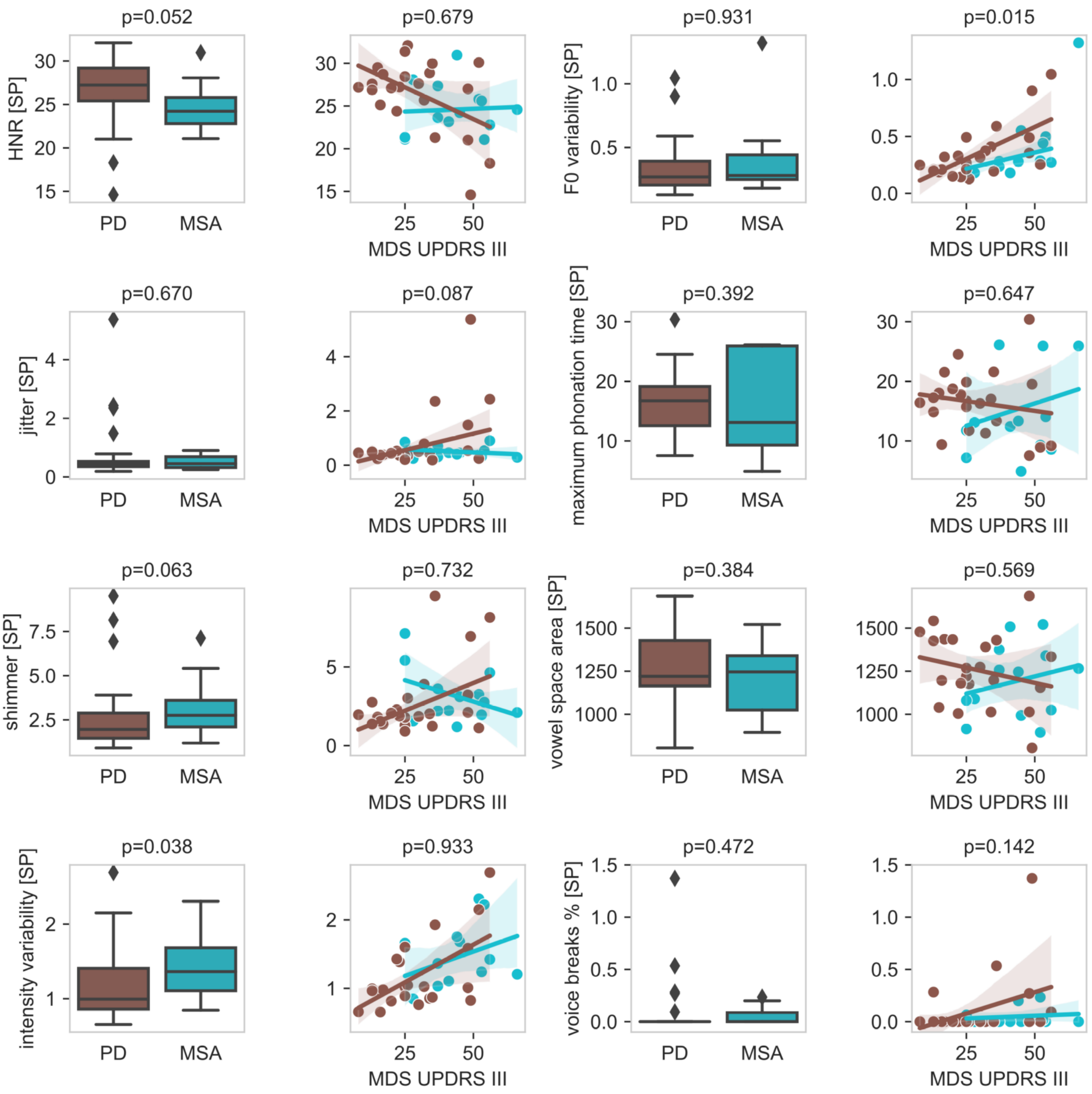

Figure S5: Speech characteristics of MSA and PD for sustained phonation tasks
The left columns show the results of univariate comparisons between speech characteristics in PD and MSA with corresponding p-values. On the right columns, the parkinsonism severity reported by MDS-UPDRS III is included in the corresponding visualization. P-values for differential diagnosis reported on the right side were corrected by MDS-UPDRS III.
Abbreviations: HNR: harmonics-to-noise ratio, SP: sustained phonation task.

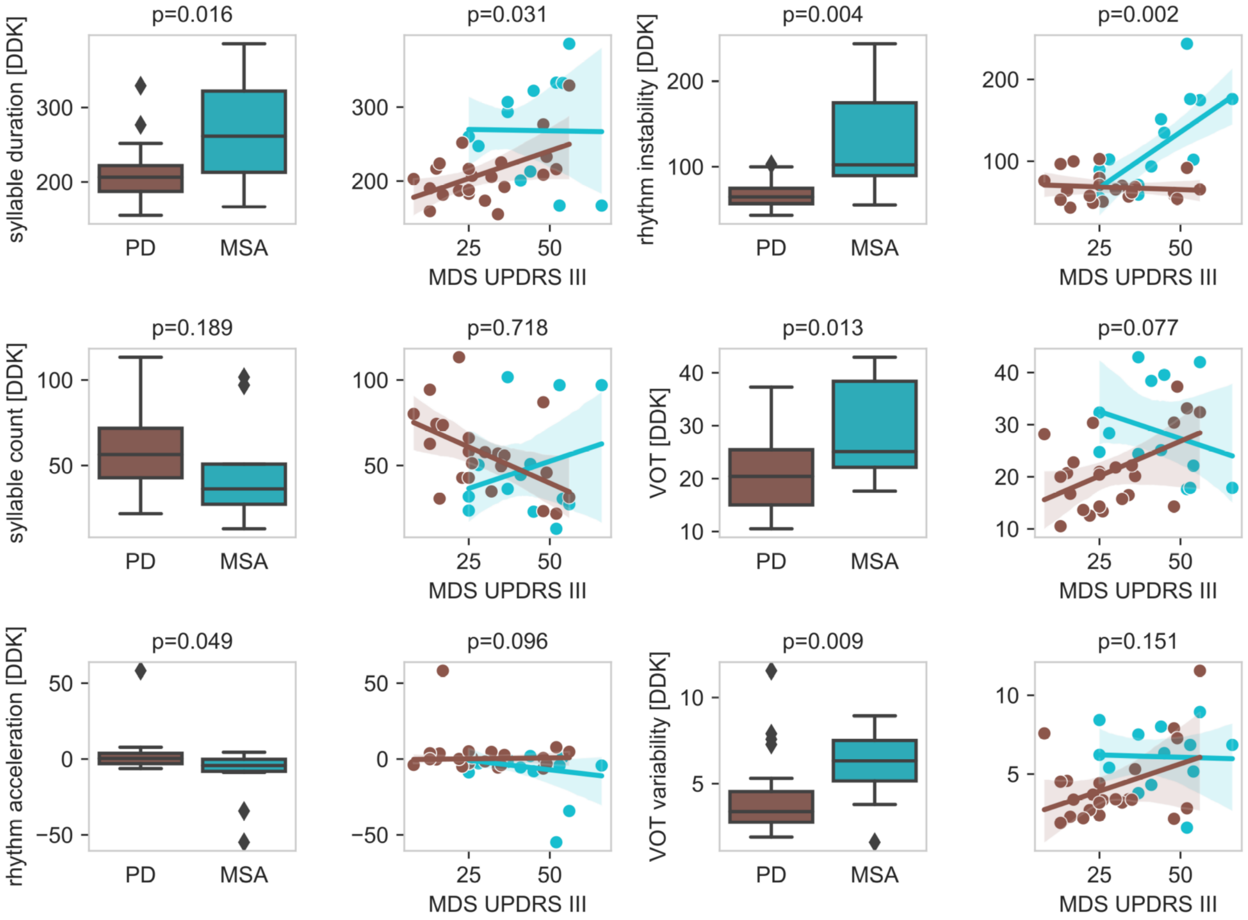

Figure S6**: **Speech characteristics of MSA and PD for diadochokinetic tasks
The left columns show the results of univariate comparisons between speech characteristics in PD and MSA with corresponding p-values. On the right columns, the parkinsonism severity reported by MDS-UPDRS III is included in the corresponding visualization. P-values for differential diagnosis reported on the right side were corrected by MDS-UPDRS III.
Abbreviations: DDK: diadochokinetic task, VOT: voice onset time.

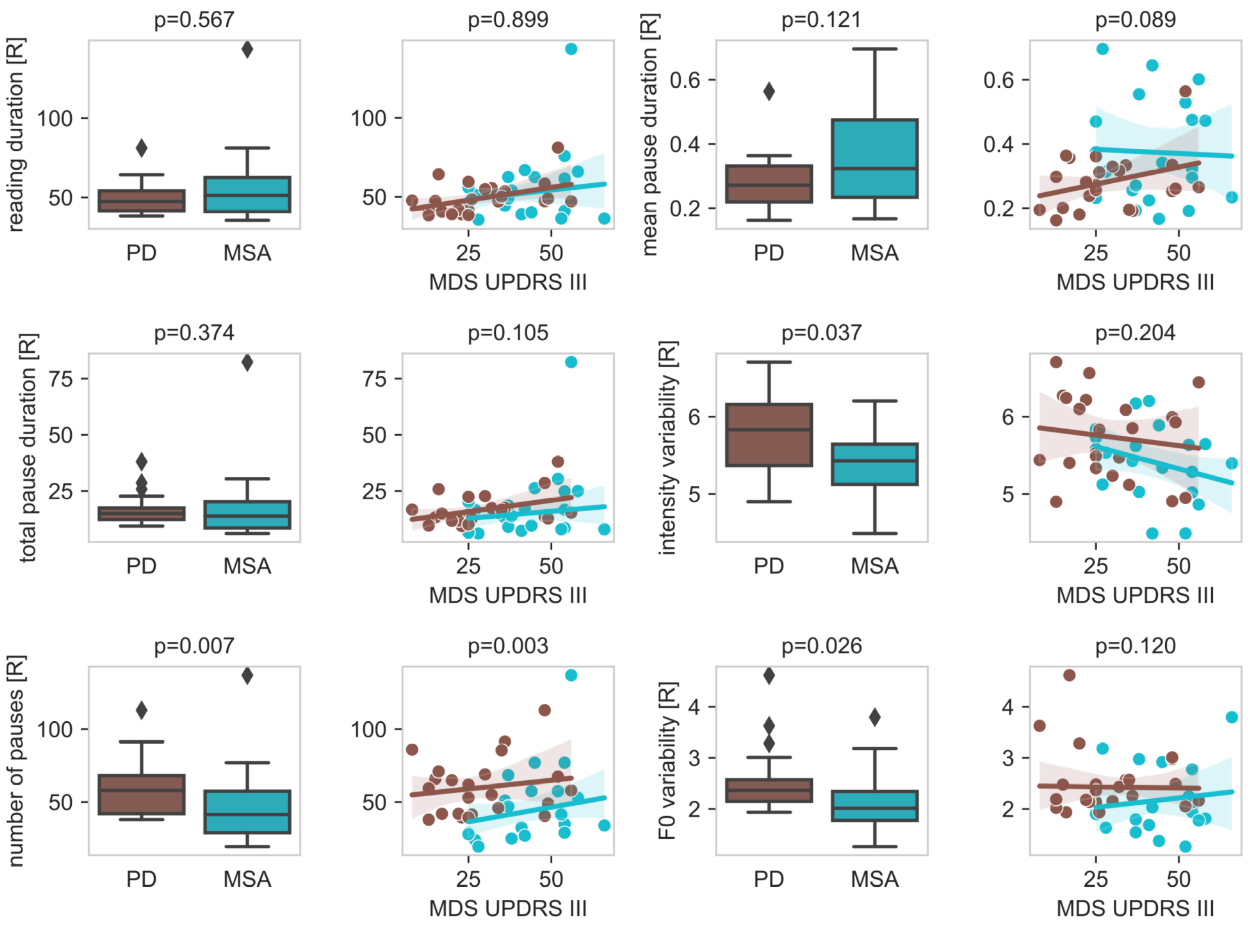

Figure S7: Speech characteristics of MSA and PD for reading task

The left columns show the results of univariate comparisons between speech characteristics in PD and MSA with corresponding p-values. On the right columns, the parkinsonism severity reported by MDS-UPDRS III is included in the corresponding visualization. P-values for differential diagnosis reported on the right side were corrected by MDS-UPDRS III.

Abbreviations: R: Reading task

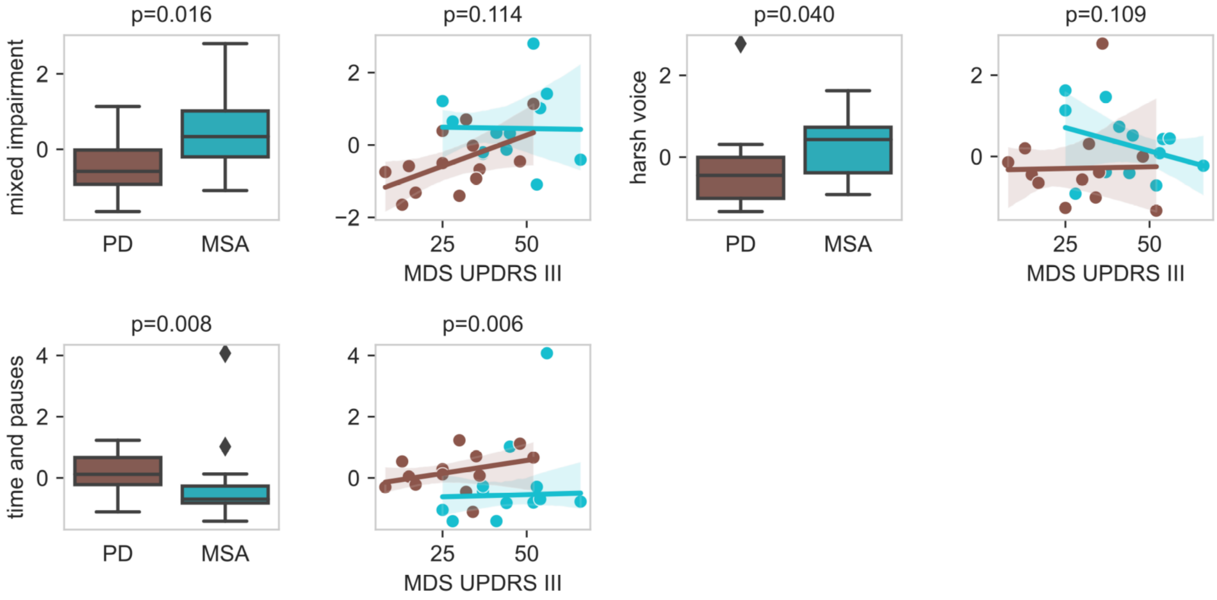

Figure S8: Speech characteristics of MSA and PD for factors of the exploratory factor analysis
The left columns show the results of univariate comparisons between the speech factors in PD and MSA with corresponding p-values. On the right columns, the parkinsonism severity reported by MDS-UPDRS III is included in the corresponding visualization. P-values for differential diagnosis reported on the right side were corrected by MDS-UPDRS III.

**Speech characteristic or speech factor**	**Speech factor**	**UMSARS correlation MSA cohort**	**MDS-UPDRS III correlation MSA cohort**	**MDS-UPDRS III correlation PD cohort**	**MSA/PD-Differences (not corrected)**	**MSA/PD-Differences (MDS-UPDRS III corrected)**
**Mean pause duration [R]**<br>PD: male > female (p<0.001)	**Mixed impairment**	p=0.883	p=0.87	p=0.12	p=0.12	p=0.09
female				p=0.08	**MSA > PD (p=0.01)**	p=0.40
male				p=0.37	p=0.20	**MSA > PD (p=0.0497)**
**Syllable count [DDK]**	**Mixed impairment**	**ρ=-0.77 (p=0.025)**	p=0.38	**ρ=-0.50 (p=0.02)**	p=0.19	p=0.72
**Syllable duration [DDK]**	**Mixed impairment**	p=0.065	p=0.96	**ρ=0.54 (p=0.010)**	**MSA > PD (p=0.02)**	**MSA > PD (p=0.03)**
**Intensity variability [SP]**	Mixed impairment	**ρ=0.71 (p=0.047)**	p=0.19	**ρ=0.59 (p=0.003)**	**MSA > PD (p=0.04)**	p=0.93
**Vowel space area [SP]**<br>PD: female > male (p=0.03)	Mixed impairment	p=0.998	p=0.42	p=0.28	p=0.38	p=0.57
female				p=0.55	p=0.30	p=0.36
male				p=0.11	p=0.48	p=0.62
**Rhythm acceleration [DDK]**	Mixed impairment	p=0.132	p=0.24	p=0.46	**PD > MSA (p=0.049)**	p=0.10
**Maximum phonation time [SP]**	Mixed impairment	p=0.607	p=0.42	p=0.44	p=0.39	p=0.65
**F0 variability [R]**	Mixed impairment	p=0.427	p=0.54	p=0.47	**PD > MSA (p=0.03)**	p=0.12
**Intensity variability [R]**	Mixed impairment	p=0.883	p=0.18	p=0.53	**PD > MSA (p=0.04)**	p=0.20
**Rhythm instability [DDK]**	Mixed impairment	p=0.199	**ρ=0.72 (p=0.005)**	p=0.63	**MSA > PD (p=0.004)**	**MSA > PD (p=0.002)**
**Mixed impairment factor**	Mixed impairment	p=0.261	p=0.95	p=0.055	**MSA > PD (p=0.02)**	p=0.11
**Total pause duration [R]**	**Time and pauses**	**ρ=0.60 (p=0.013)**	p=0.17	p=0.05	p=0.37	p=0.10
**Number of pauses [R]**<br>PD: female > male (p<0.001)	**Time and pauses**	**ρ=0.63 (p=0.010)**	p=0.11	p=0.45	**PD > MSA (p=0.007)**	**PD > MSA (p=0.003)**
female				**ρ=0.78 (p=0.04)**	**PD>MSA (p=0.001)**	**PD > MSA (p<0.001)**
male				p=0.38	**PD>MSA (p=0.02)**	**PD > MSA (p=0.01)**
**Reading duration [R]**	**Time and pauses**	**ρ=0.62 (p=0.011)**	p=0.16	**ρ=0.44 (p=0.03)**	p=0.57	p=0.90
**Voice onset time (VOT) [DDK]**	Time and pauses	p=0.231	p=0.35	**ρ=0.49 (p=0.02)**	**MSA > PD (p=0.01)**	p=0.08
**Time and pauses factor**	Time and pauses	p=0.369	p=0.34	p=0.25	**PD > MSA (p=0.008)**	**PD > MSA (p=0.006)**
**Shimmer [SP]**	**Harsh voice**	p=0.848	p=0.16	**ρ=0.49 (p=0.02)**	p=0.06	p=0.73
**Harmonics-to-noise ratio (HNR) [SP]**	**Harsh voice**	p=0.498	p=0.85	**ρ=-0.48 (p=0.02)**	p=0.05	p=0.68
**Jitter [SP]**	**Harsh voice**	p=0.814	p=0.41	**ρ=0.53 (p=0.010)**	p=0.67	p=0.09
**VOT variability [DDK]**	Harsh voice	p=0.141	p=0.91	p=0.05	**MSA > PD (p=0.009)**	p=0.15
**Voice breaks % [SP]**	Harsh voice	p=0.837	p=0.64	p=0.08	p=0.47	p=0.14
**F0 variability [SP]**	Harsh voice	p=0.156	**ρ=0.66 (p=0.01)**	**ρ=0.67 (p=0.001)**	p=0.93	**PD > MSA (p=0.01)**
**Harsh voice factor**	Harsh voice	p=0.791	p=0.23	p=0.95	**MSA > PD (p=0.04)**	p=0.11

Table S3: Summary of speech characteristics findings.

Summary of speech factor compositions, MDS-UPDRS III and UMSARS correlations, and differences between PD and MSA for all speech characteristics. Significance of differences between PD and MSA are shown with and without MDS-UPDRS III correction. Corresponding speech factors are shown in bold if the speech characteristic was considered relevant for the speech factor (i.e., factor loading greater than 0.63). Significant correlation coefficients and differences between MSA and PD are shown in bold. Pearson correlation coefficients are reported for significant correlations. Results of sex-specific subgroup analyses are shown for speech characteristics with significant differences between male and female patients.

Abbreviations: DDK: diadochokinetic task, HNR: harmonics-to-noise ratio, R: reading task, SP: sustained phonation task, VOT: voice onset time.

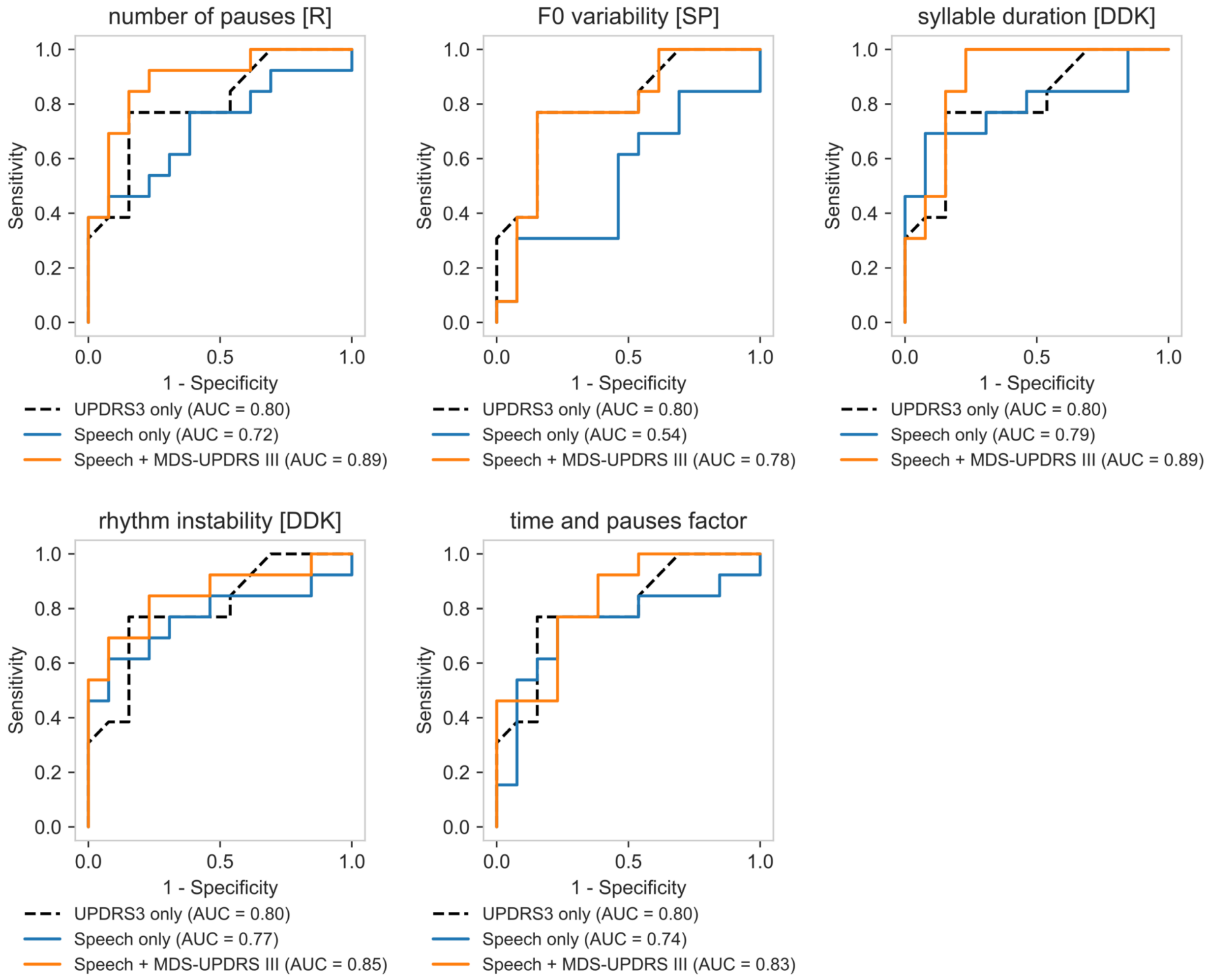

Figure S9: ROC curves for differential diagnosis between MSA and PD.

Comparison of receiver operator characteristic (ROC) curves for different logistic regression models. All models are compared with a null model including only the MDS-UPDRS III and no speech characteristic (black dashed line). Models including only one speech characteristic and no MDS-UPDRS III are depicted in blue. Models including one speech characteristic and MDS-UPDRS III are depicted in orange. AUC-ROC scores are shown in the legend. The speech characteristic or speech factor which was used is indicated above the plot.

Abbreviations: DDK: diadochokinetic task, R: reading task, SP: sustained phonation task.

Speech tasks

For sustained vowel phonation, the subjects had to produce a sustained phonation of the vowel /a/ and /i/ for as long and steadily as possible. For alternating/sequential motion rates, subjects were instructed to repeat the syllables as accurately and quickly as possible within one breath. For text reading, subjects read the German linguistic text "Nordwind und Sonne" (4) at a comfortable loudness and speed.

Reading text (German)

Einst stritten sich Nordwind und Sonne, wer von ihnen beiden wohl der Stärkere wäre, als ein Wanderer, der in einen warmen Mantel gehüllt war, des Weges daherkam. Sie wurden einig, dass derjenige für den Stärkeren gelten sollte, der den Wanderer zwingen würde, seinen Mantel abzunehmen. Der Nordwind blies mit aller Macht, aber je mehr er blies, desto fester hüllte sich der Wanderer in seinen Mantel ein. Endlich gab der Nordwind den Kampf auf. Nun erwärmte die Sonne die Luft mit ihren freundlichen Strahlen, und schon nach wenigen Augenblicken zog der Wanderer seinen Mantel aus. Da musste der Nordwind zugeben, dass die Sonne von ihnen beiden der Stärkere war.

Reading text (translated)

Once upon a time, the North Wind and the Sun were arguing about which of them was the stronger when a wanderer came along the path wrapped in a warm cloak. They agreed that the one who would force the hiker to take off his coat would be considered the strongest. The north wind blew with all its might, but the harder it blew, the tighter the hiker wrapped himself in his coat. Finally, the north wind gave up the fight. Now the sun warmed the air with its friendly rays, and after just a few moments the hiker took off his coat. The north wind had to admit that the sun was the stronger of the two of them.

Acoustic analysis

The sound intervals used for formant measurements were chosen manually by visual inspection in Praat. Furthermore, all acoustic measurements were visually inspected in Praat and parameters were individually adjusted for each patient if the default parameters performed insufficiently. The default parameters used for the Praat routines are listed below. *Intensity variability*, *F0*, *F0 variability*, *HNR*, *jitter* and *shimmer* were calculated after removing the first and last 10% of the sustained phonation recording as recommended (2). Furthermore, *intensity variability, F0* and *F0 variability* were calculated after removing pauses from the text reading recordings (3). VOTs were measured based on visual inspection of raw waveform and wide-band spectrogram from the initial stop burst to the onset of periodicity associated with the vowel (5).

The speech characteristics (1) *reading duration*, (2) *maximum phonation time*, (3) *syllable duration*, (4) *syllable count*, (5) *rhythm acceleration*, (6) *rhythm instability*, (7) *voice onset time (VOT)* and (8)

VOT variability were calculated based on manual inspection and annotation of the sound files in Praat. For robust measurements to outliers, we used the median syllable length, median VOT and median absolute deviation of VOTs. Rhythm acceleration and instability were calculated by modeling a linear increase or decrease in speech rhythm (3).

To increase the robustness of our measurements and to limit the number of speech characteristics to a reasonable number, the following steps were taken: All speech tasks and calculations described above were performed twice, and only the means of both recordings were used. For sustained phonation, the means of the speech characteristics from the /a/ and /i/ tasks were calculated. Also, means of the speech characteristics of /pΛ/, /tΛ/ and /pΛtΛkΛ/ were calculated.

To account for potential sex differences in speech, the following steps were taken: *F0* was analyzed separately for male and female patients due to the higher pitch in females (Fig. S2). For all other speech characteristics, male and female patients were initially analyzed together. Speech characteristics that showed significant differences between males and females in MSA or PD were subsequently analyzed in sex-specific subgroups (Table S3).

Default Praat parameters

The complete Praat scripts including all parameters can be obtained from our github repository: github.com/t-haehnel/MSA-Speech-Analysis-Praat.

In general, we used sex-specific settings for pitch and formants:

male:

- minimum pitch: 60 Hz
- maximum pitch: 300 Hz
- formant ceiling: 4000 Hz

female:

- minimum pitch: 100 Hz
- maximum pitch: 500 Hz
- formant ceiling: 6000 Hz

The following parameters were used for **both** female and male:

- number of formants: 4
- formant window: 0.04 s
- minimum silence time for pause detection: 60 ms

All results were checked by visual inspection in Praat and default parameters were adjusted if needed.

Statistical analyses

Exploratory factor analysis using a minimum residual solution and no factor rotation was performed on MSA and PD patients with complete recordings, based on all speech characteristics except *F0*, as this parameter is highly influenced by sex. To account for the smaller number of MSA patients who completed all tasks, we selected an age- and sex-matched subgroup of the PD cohort of equal size for the exploratory factor analysis. The number of three factors was determined visually from the scree plot (Fig. S3).

To compare speech characteristics between patients with multiple system atrophy (MSA) and Parkinson’s disease (PD), we employed a linear model while accounting for motor impairment. Specifically, we included the MDS-UPDRS III score as a covariate to control for motor symptom severity. The following linear model was used for statistical analysis:

$$Y = \beta_0 + \beta_1 * \text{Group} + \beta_2 * \text{MDS-UPDRS III}$$

where:

- Y represents the speech characteristic of interest,
- Group is a categorical variable indicating diagnosis (MSA or PD),
- MDS-UPDRS III is included as a covariate to adjust for motor impairment,
- β_0,β_1, and β_2 are the model coefficients

This approach ensures that observed differences in speech characteristics between MSA and PD are not confounded by differences in motor severity as assessed by the MDS-UPDRS III. The significance level of β_1 is reported within the manuscript and the supplementary figures.

To explore the potential for discriminating MSA from PD, logistic regression models using different predictors were analyzed: (I) a null model including only MDS-UPDRS III, (II) models including only a single speech characteristic or speech factor, and (III) models including MDS-UPDRS III and either one speech characteristic or one speech factor. Only speech characteristic with significant differences between MSA and PD after correcting for MDS-UPDRS III were considered.

All tests were conducted as two-tailed with a significance level 0.05. Statistical analyses were performed using Python 3.10.8 (6) with the packages scipy 1.9.3 (7), factor-analyzer 0.4.1 (8), pingouin 0.5.2 (9), statsmodels 0.13.2 (10), and scikit-learn 1.0.2 (11).

Detailed discussion of MSA and PD speech characteristics compared to previous literature

Similar differences between MSA and PD have been reported by others for *F0 variability* in the reading task (12), *intensity variability* in the sustained phonation task (13), *syllable duration* (12,14), *rhythm instability* (3,12), *VOT* (12) and *VOT variability* (14), showing more severe speech impairment in the MSA cohort when compared to PD. Furthermore, our results are consistent with previous findings that male MSA patients have higher F0 than male PD patients (15) and suggest that F0 may also be higher in female MSA patients compared to female PD patients. We observed less *F0 variability* in the sustained phonation task in MSA when correcting for MDS-UPDRS III, whereas several studies reported higher impairment in MSA compared to PD in an MDS-UPDRS III uncorrected analysis (12,13,16). Furthermore, we found a higher *rhythm acceleration* and higher *number of pauses* in PD patients compared to MSA, which has not been reported before.

Speech characteristics have been extensively studied as a marker of disease progression in PD (1,17–19). Longitudinal studies have shown that markers of rhythm instability and rhythm acceleration (17), *vowel space area* and *vowel articulation index* (18), *shimmer, HNR,* and several pause characteristics show significant changes with PD progression (19). In contrast, whether speech characteristics are directly correlated with motor impairment as reported by UPDRS III remains controversial. While some studies reported significant correlations of prosodic parameters (20,21), several studies found no relationship with motor impairment (17–20,22,23). Our results support previous findings (20,21) that *jitter, shimmer, HNR,* and *F0 variability* of the sustained phonation task report motor impairment. Beyond these known associations, we show that *intensity variability* of the sustained phonation task, *syllable duration*, *syllable count*, *reading duration and VOT* also capture motor impairment.

Previous publications on MSA speech distinguished three domains of speech impairment based on neuropathological constructs and perception: hypokinetic, ataxic, and spastic (12,16,24,25). In general, our study did not confirm this “syndromal” classification from a data-driven perspective. Hypokinetic, ataxic, and spastic characteristics were distributed along all three speech factors and did not show an association with any of them. For example, *reading duration* and *syllable duration* are typically considered spastic speech characteristics. However, reading duration is part of the *time and pauses* factor, while syllable duration is part of the *mixed speech characteristics* factor. Similarly, hypokinetic speech characteristics appear in all three speech factors (e.g., *number of pauses – time and pauses* factor, *shimmer* and *jitter – harsh voice* factor, *mean pause duration – mixed speech characteristics* factor).

Furthermore, most of the speech characteristics traditionally interpreted as hypokinetic characteristics showed no correlation with MDS-UPDRS III in PD patients (i.e., *number of pauses*, *mean pause duration*, *F0 variability of reading task*, *maximum phonation time*, *vowel space area*, *rhythm acceleration*). In MSA, none of the traditional hypokinetic speech characteristic correlated with MDS-UPDRS III. In contrast, two traditional ataxic speech characteristics (*F0 variability* of sustained phonation and *rhythm instability*) correlated with MDS-UPDRS III.

References

1. Brabenec L, Mekyska J, Galaz Z, Rektorova I. Speech disorders in Parkinson’s disease: early diagnostics and effects of medication and brain stimulation. J Neural Transm (Vienna). 2017 Mar;124(3):303–34.
2. Rusz J, Tykalova T, Ramig LO, Tripoliti E. Guidelines for Speech Recording and Acoustic Analyses in Dysarthrias of Movement Disorders. Mov Disord. 2021 Apr;36(4):803–14.
3. Rusz J, Hlavnička J, Čmejla R, Růžička E. Automatic Evaluation of Speech Rhythm Instability and Acceleration in Dysarthrias Associated with Basal Ganglia Dysfunction. Front Bioeng Biotechnol. 2015;3:104.
4. International Phonetic Association, editor. Handbook of the International Phonetic Association: a guide to the use of the International Phonetic Alphabet. Cambridge, U.K. ; New York, NY: Cambridge University Press; 1999. 204 p.
5. Fischer E, Goberman AM. Voice onset time in Parkinson disease. Journal of Communication Disorders. 2010 Jan;43(1):21–34.
6. Python Software Foundation. Python Language Reference [Internet]. Available from: https://www.python.org
7. SciPy 1.0 Contributors, Virtanen P, Gommers R, Oliphant TE, Haberland M, Reddy T, et al. SciPy 1.0: fundamental algorithms for scientific computing in Python. Nat Methods. 2020 Mar;17(3):352–352.
8. Biggs J. factor-analyzer. A Factor Analysis tool written in Python. [Internet]. Available from: https://github.com/EducationalTestingService/factor_analyzer
9. Vallat R. Pingouin: statistics in Python. JOSS. 2018 Nov 19;3(31):1026.
10. Seabold S, Perktold, Josef. statsmodels: Econometric and statistical modeling with python. In: 9th Python in Science Conference. 2010.
11. Pedregosa F, Varoquaux G, Gramfort A, Michel V, Thirion B, Grisel O, et al. Scikit-learn: Machine Learning in Python. Journal of Machine Learning Research. 2011;12:2825–30.

12. Rusz J, Tykalová T, Salerno G, Bancone S, Scarpelli J, Pellecchia MT. Distinctive speech signature in cerebellar and parkinsonian subtypes of multiple system atrophy. J Neurol. 2019 Jun;266(6):1394–404.
13. Kowalska-Taczanowska R, Friedman A, Koziorowski D. Parkinson's disease or atypical parkinsonism? The importance of acoustic voice analysis in differential diagnosis of speech disorders. Brain Behav. 2020 Aug;10(8):e01700.
14. Tykalova T, Rusz J, Klempir J, Cmejla R, Ruzicka E. Distinct patterns of imprecise consonant articulation among Parkinson's disease, progressive supranuclear palsy and multiple system atrophy. Brain Lang. 2017 Feb;165:1–9.
15. Huh YE, Park J, Suh MK, Lee SE, Kim J, Jeong Y, et al. Differences in early speech patterns between Parkinson variant of multiple system atrophy and Parkinson's disease. Brain Lang. 2015 Aug;147:14–20.
16. Rusz J, Bonnet C, Klempíř J, Tykalová T, Baborová E, Novotný M, et al. Speech disorders reflect differing pathophysiology in Parkinson's disease, progressive supranuclear palsy and multiple system atrophy. J Neurol. 2015;262(4):992–1001.
17. Skodda S, Flasskamp A, Schlegel U. Instability of syllable repetition as a marker of disease progression in Parkinson's disease: A longitudinal study: Instability of Syllable Repetition. Mov Disord. 2011 Jan;26(1):59–64.
18. Skodda S, Grönheit W, Schlegel U. Impairment of vowel articulation as a possible marker of disease progression in Parkinson's disease. PLoS One. 2012;7(2):e32132.
19. Skodda S, Grönheit W, Mancinelli N, Schlegel U. Progression of voice and speech impairment in the course of Parkinson's disease: a longitudinal study. Parkinsons Dis. 2013;2013:389195.
20. Suphinnapong P, Phokaewvarangkul O, Thubthong N, Teeramongkonrasmee A, Mahattanasakul P, Lorwattanapongsa P, et al. Objective vowel sound characteristics and their relationship with motor dysfunction in Asian Parkinson's disease patients. J Neurol Sci. 2021 Jul 15;426:117487.

21. Tsanas A, Little MA, McSharry PE, Ramig LO. Accurate Telemonitoring of Parkinson’s Disease Progression by Noninvasive Speech Tests. IEEE Trans Biomed Eng. 2010 Apr;57(4):884–93.
22. Skodda S, Rinsche H, Schlegel U. Progression of dysprosody in Parkinson’s disease over time-A longitudinal study. Mov Disord. 2009 Apr 15;24(5):716–22.
23. Rusz J, Hlavnička J, Tykalová T, Bušková J, Ulmanová O, Růžička E, et al. Quantitative assessment of motor speech abnormalities in idiopathic rapid eye movement sleep behaviour disorder. Sleep Med. 2016 Mar;19:141–7.
24. Daoudi K, Das B, Tykalova T, Klempir J, Rusz J. Speech acoustic indices for differential diagnosis between Parkinson’s disease, multiple system atrophy and progressive supranuclear palsy. npj Parkinsons Dis. 2022 Oct 27;8(1):142.
25. Kluin KJ, Gilman S, Lohman M, Junck L. Characteristics of the Dysarthria of Multiple System Atrophy. Archives of Neurology. 1996 Jun 1;53(6):545–8.
