# Supplementary figures and images for: Speech Differences between Multiple System Atrophy and Parkinson's Disease
Source: Mov Disord Clin Pract. 2025 May 3;12(9):1391–6. doi: 10.1002/mdc3.70094 (PMC12481427; doi:10.1002/mdc3.70094)

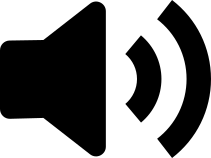

Supplement: Supplementary file 1 — File S1. The file contains additional figures and text presenting methodological details, definitions and explanations of speech characteristics, details of speech tasks and acoustic analysis, the default Praat parameters, speech factor analysis, and sex‐specific analyses. File S2. Audio file of the text reading task from a MSA patient presenting a mixed dysarthria type characterized by hypokinetic (hypophonia, reduced articulation precision, prolonged pauses), ataxic (voice breaks, pitch fluctuations) and spastic/dystonic characteristics (high pitch). The German text and its English translation is given in Supplemental File S1. File S3. Audio file of the text reading task from a PD patient presenting a hypokinetic dysarthria type characterized by typical hypokinetic speech characteristics (hypophonia, reduced articulation precision, prolonged pauses). The German text and its English translation is given in Supplemental File S1. TABLE S1. List of speech characteristics calculated for the different speech tasks. TABLE S2. Clinical characteristics of the MSA and PD cohorts. TABLE S3. Summary of speech characteristics findings. [file MDC3-12-1391-s001.zip › mdc370094-supitem-0001/audio.png]
